# Hybrid Nanobeads for Oral Indomethacin Delivery

**DOI:** 10.3390/pharmaceutics14030583

**Published:** 2022-03-08

**Authors:** Flávia Monique Rocha Bonetti, Eneida de Paula, Belchiolina Beatriz Fonseca, Gabriela Ribeiro da Silva, Leandro Santana Soares da Silva, Ludmilla David de Moura, Márcia Cristina Breitkreitz, Gustavo Henrique Rodrigues da Silva, Lígia Nunes de Morais Ribeiro

**Affiliations:** 1 Department of Biochemistry and Tissue Biology, Institute of Biology, University of Campinas—UNICAMP, Campinas 13083-862, SP, Brazil; flaa.monique@hotmail.com (F.M.R.B.); depaula@unicamp.br (E.d.P.); ludmilladavidm@gmail.com (L.D.d.M.); gustavohrs@gmail.com (G.H.R.d.S.); 2 School of Veterinary Medicine, Federal University of Uberlândia—UFU, Uberlândia 38410-337, MG, Brazil; biafonseca@ufu.br (B.B.F.); gabirs.05@gmail.com (G.R.d.S.); leandrosantana.1522@gmail.com (L.S.S.d.S.); 3 Department of Analytical Chemistry, Institute of Chemistry, University of Campinas—UNICAMP, Campinas 13083-970, SP, Brazil; marciacb@unicamp.br; 4 Institute of Biotechnology, Federal University of Uberlândia—UFU, Uberlândia 38405-302, MG, Brazil

**Keywords:** NLC, hybrid nanobead, biopolymers, anti-inflammatory, chicken embryo model

## Abstract

The oral administration of the anti-inflammatory indomethacin (INDO) causes severe gastrointestinal side effects, which are intensified in chronic inflammatory conditions when a continuous treatment is mandatory. The development of hybrid delivery systems associates the benefits of different (nano) carriers in a single system, designed to improve the efficacy and/or minimize the toxicity of drugs. This work describes the preparation of hybrid nanobeads composed of nanostructured lipid carriers (NLC) loading INDO (2%; *w*/*v*) and chitosan, coated by xanthan. NLC formulations were monitored in a long-term stability study (25 °C). After one year, they showed suitable physicochemical properties (size < 250 nm, polydispersity < 0.2, zeta potential of −30 mV and spherical morphology) and an INDO encapsulation efficiency of 99%. The hybrid (lipid-biopolymers) nanobeads exhibited excellent compatibility between the biomaterials, as revealed by structural and thermodynamic properties, monodisperse size distribution, desirable in vitro water uptake and prolonged in vitro INDO release (26 h). The in vivo safety of hybrid nanobeads was confirmed by the chicken embryo (CE) toxicity test, considering the embryos viability, weights of CE and annexes and changes in the biochemical markers. The results point out a safe gastro-resistant pharmaceutical form for further efficacy assays.

## 1. Introduction

Indomethacin (INDO) is a non-steroidal anti-inflammatory (NSAID) agent widely used for pain, fever and inflammation control through oral administration [1]. However, severe side effects in the gastrointestinal system (e.g., nausea, indigestion, vomit, diarrhea and abdominal ache) are associated to the oral administration, due to the first-pass metabolism. These effects are exacerbated in chronic diseases, in which an extended treatment is required [2]. Considering the huge number of INDO prescriptions (>1 million/per year, only in the USA), new approaches to minimize INDO systemic toxicity and improve its efficacy are required [3].

Colloidal drug delivery systems (DDS) with optimized therapeutic actions should decrease side effects by the sustained release of the loaded drugs [4]. Nanostructured lipid carriers (NLC), the second generation of lipid nanoparticles, are formed by a mixture of two or more lipids (solid and liquid at room temperature) and a surfactant [5]. NLC formulations loading different anti-inflammatory compounds for multi-application and administration routes have been successfully reported [6,7,8]. However, as a colloid, NLC cannot provide suitable gastro-resistance and mucoadhesion [9], as required by the oral route.

Lipid–polymer nanohybrid formulations combine the benefits of each carrier/excipient in a single DDS. Nanohybrid pharmaceuticals should have at least one nanostructured material in the composition [10]. Such systems are molecularly planned to achieve specialized interactions with the target, that are specific for each purpose [11]. Biopolymers are natural, versatile, biocompatible, cheap and biodegradable matrices widely used as DDS [12]. Chitosan (CHT) is a cationic polysaccharide with remarkable mucoadhesive properties, obtained from the chitin deacetylation of crustacea shells. The available amine groups of CHT can electrostatically interact with the anionic mucin from mucous tissues, increasing the CHT residence time [13]. However, CHT is sensitive to acid media, such as the gastric microenvironment [14]. On the other hand, xanthan gum (XAN) is an anionic exopolysaccharide secreted by *Xanthomonas campestris* [15]. Currently, XAN is processed by different forms, acting as rheological improver, thickener and coating agent in the food, cosmetic and pharmaceutical industries [16].

In this work, two different strategies (hybridization and coating) were combined in order to decrease the toxicity and prolong the INDO release profile, simulating oral administration. A novel lipid–biopolymer nanobead was developed with NLC (composed of myristyl myristate, coconut oil and poloxamer) encapsulating INDO as the lipid excipient blended with CHT solution, and coated by XAN. The structural compatibility between NLC/INDO and the biopolymers provided well-designed coated-based nanobeads. The resultant nanobeads slowed suitable in vitro INDO release in the first 2 h of the experiment (mimicking the gastric medium at pH 1.2) and prolonged the subsequent release for 26 h in a medium (pH 6.8) simulating the intestinal site. XAN-coated nanobeads provided gastro-resistance to the system, preventing CHT and NLC/INDO solubilization in a simulated gastric medium, allowing for CHT mucoadhesive activity at the intestinal mucosa. The safety of NLC and nanobeads was confirmed by in vitro (in HaCat cells in culture) and in vivo (chicken embryo model). The resultant hybrid nanobead is as an excellent candidate for the oral delivery of INDO, as well as other anti-inflammatories.

## 2. Materials and Methods

### 2.1. Materials

Pluronic^®^ 68 (P68), indomethacin (INDO), xanthan gum (XAN), chitosan (CHT) and 3-(4,5-dimethylthiazol-2-yl)-2,5-diphenyltetrazolium bromide (MTT) were supplied by Sigma (St. Louis, MI, USA). Myristyl myristate (MM) was provided by Dhaymers Química Fina (Taboão da Serra, SP, Brazil) and coconut oil (CO) was purchased from Engenharia das Essências, LTDA (São Paulo, SP, Brazil). Deionized water (18 MΩ) was obtained from an Elga USF Maxima Ultra-Pure water purifier (Elga, Lane End, UK).

### 2.2. Preparation of NLC Formulations Loading Indomethacin

NLC (as control formulation) and NLC-INDO (2%, *w*/*v*) were prepared by the ultrasonication emulsification method [17]. The composition and concentration of the excipients used in NLC are displayed in Table 1: myristyl myristate (MM) as the solid lipid, coconut oil (CO) as the liquid lipid and poloxamer as the surfactant. The total lipid (TL) concentration—the sum of solid and liquid lipid concentrations—was kept in 10% (*w*/*v*). Briefly, the lipid phase (containing MM and CO), with or without INDO (2%), was heated 10 °C above the solid lipid melting point (~48 °C) under magnetic stirring [18]. The aqueous phase composed of P68 (2–5%) was heated at the same temperature and dropped into the lipid phase under high-speed homogenization (10,000 rpm) for 3 min in an Ultra-Turrax blender (IKA Werke, Staufen, Germany). The resultant microemulsion was tip sonicated (Vibra-Cell, Sonics & Materials Inc., Newtown, CT, USA) at 500 W and 20 kHz, in 30 s (on/off) cycles for 20 min and further cooled to 25 °C in an ice bath.

### 2.3. Structural Characterization

The structural characterization of NLC and NLC/INDO (2%) was performed by dynamic light scattering (DLS), in terms of particles size (nm) and polydispersity index (PDI). Zeta potential (mV) values were obtained by electrophoretic light scattering in the same Zetasizer Nano ZS90 (Malvern Instruments, Worcestershire, UK) equipment. The pH values of NLC were determined with a (Tecnal, Uberlândia, Brazil) pH meter. The results were expressed as means ± standard deviations (*n* = 3).

### 2.4. Long-Term Physicochemical Stability

A long-term stability study of NLC and NLC/INDO (2%), stored in Falcon tubes and maintained at room temperature for one year (25 °C), monitored the particle size (nm), PDI, Zeta potential (mV) and pH values, in triplicates. ANOVA/ Tukey tests were employed to elucidate intragroup statistically differences over time (*α* = 0.05).

### 2.5. Transmission Electron Microscopy

The morphological analyses of NLC and NLC/INDO (2%) samples were performed by transmission electron microscopy (TEM). The samples were diluted 50×, dispersed onto copper grids coated with a carbon film and dried at 25 °C. Uranyl acetate (2%) was added to provide contrast. After 24 h, micrographs of the samples were obtained using a JEOL 1200 EXII (Jeol, Peabody, MA, USA) microscope, at 60 kV.

### 2.6. Determination of Indomethacin Encapsulation Efficiency

INDO calibration curve by UV-vis at 210 nm was performed on five different concentrations in the range of 3.25–20 µg/mL (r^2^ > 0.99). Each point represents the average of 9 measurements performed on 3 different days (Figure S1 (Supplementary Materials)). INDO encapsulation efficiency (%*EE*) was quantified by the ultrafiltration–centrifugation method [9], using regenerated cellulose filters with a molecular exclusion pore size of 10 kDa (Millipore, Burlington/MA, EUA). The samples (triplicates) were diluted and centrifuged at 4000× *g* for 20 min. The total amount (*INDO_total_*) and the non-encapsulated indomethacin (*INDO_free_*) present in the filtered fraction were quantified by UV-vis (Waters, Agilent Technologies, Milford, MA, USA) at 210 nm [19] for the determination of the %*EE* value, according to Equation (1):

(1) $$\%EE=\frac{INDO_{total}-INDO_{free}}{INDO_{total}}\times 100$$

### 2.7. In Vitro Cell Viability Test

The in vitro cytotoxicity of INDO in solution (control) and NLC/INDO were determined by the MTT assay in HaCat keratinocytes purchased from Federal University of Rio de Janeiro cells bank, Federal University of Rio de Janeiro, Brazil. Briefly, the cells were seeded in 96-well culture plates at a density 10^4^ cells/mL and incubated for 24 h at 37 °C under 5% CO_2_. The RPMI culture medium was then discarded and replaced with 100 μL of fresh medium containing increasing concentrations of INDO or NLC/INDO. After 24 h, the medium was removed, and the plate was washed with phosphate-buffered saline, at pH 7.4. After that, 100 μL of medium (without serum) containing 0.5 mg/mL of MTT supplemented each well, which was then incubated for 3 h at 37 °C. The MTT solution was then removed, and 100 μL of ethanol was added to solubilize the formed formazan crystals. The formazan absorbance was measured in a microplate reader, at 570 nm [20]. The obtained absorbance was normalized to % of control. The results were provided as means ± standard deviation (SD), *n* = 9. The statistical analyses were performed by a t test (*α* = 0.05).

### 2.8. Preparation of Biopolymers and Nanobeads Loading Indomethacin

Chitosan (CHT) was dispersed in 50 mL of 0.1% acetic acid, and xanthan (XAN) was dissolved in 50 mL of deionized water under magnetic stirring until complete homogenization, with a final concentration of 2% and 0.5% (*w*/*v*), respectively. Then, 2% hydroalcoholic INDO solution was added to the CHT solution and stirred for 2 h at room temperature (CHT/INDO). For the preparation of hybrid nanobeads, NLC loading 2% INDO was used to replace half of the acid solution for CHT solubilization, and the samples were stirred for 2 h at room temperature (CHT/NLC-INDO). The total proportion of NLC/INDO:CHT in the formulations was 1:1 (*v*/*v*).

The resultant dispersions of CHT/INDO and CHT/NLC-INDO were then placed in a burette and dropped into a solution of 5% sodium tripolyphosphate and gently stirred for 15 min. The resulting CHT/INDO bead and CHT/NLC-INDO nanobead (given the nanostructured lipid excipient) were filtered, washed 3 times with abundant deionized water, frozen in liquid nitrogen and lyophilized in a (Cryodos, Telstar, Spain) freeze-dryer for subsequent analyses.

For the preparation of the final (XAN-coated) forms, aliquots of prepared CHT/INDO (bead) and CHT/NLC-INDO (nanobead) were immersed in 0.5% (*w*/*v*) XAN aqueous solution, filtered, frozen at −20 °C and lyophilized in a freeze-drier as mentioned above, resulting in the coated-based beads (XAN@CHT/INDO) and nanobeads (XAN@CHT/NLC-INDO). The INDO solution was incorporated directly in CHT solution to reach a final INDO concentration of 2% (*w*/*v*) in both hybrid nanobeads. Digital photos of the resultant beads (CHT/INDO and XAN@CHT/INDO) and nanobeads (CHT/NLC-INDO and XAN@CHT/NLC-INDO) were obtained and analyzed with the ImageJ software for the particle size distribution estimation. Table 2 displays the composition of the resultant beads and nanobeads loading INDO (2%).

### 2.9. FE-SEM Analyses of Beads and Nanobeads

The surface analysis of beads and nanobeads was carried out in a field emission scanning electron microscopy (FE-SEM) (FEI-NOVA NanoSEM 230, Sydney, Australia). XAN@CHT/NLC-INDO nanobeads were firstly frozen in liquid nitrogen and cross-sectioned with a scalpel. The samples were fixed on a carbon tape and subjected to a gold conducive coating on the surface.

### 2.10. Differential Scanning Calorimetry

Differential scanning calorimetry (DSC) analyses were carried out in a TA Q20 calorimeter (TA Instruments, New Castle, DE, USA) equipped with a cooling system of INDO, NLC excipients and nanobeads (XAN@CHT/NLC-INDO). The samples (5 mg) were introduced in aluminum pans and the thermograms registered from 25 to 180 °C, at 10 °C/min heating rate, under N_2_ flow.

### 2.11. In Vitro Water Uptake of Beads and Nanobeads

The swelling properties of beads (CHT/INDO and XAN@CHT/INDO) and nanobeads (CHT/NLC-INDO and XAN@CHT/NLC-INDO) were determined. The samples (0.05 g) were placed in Petri dishes and immersed in 10 mM phosphate solution at pH 1.2 or phosphate buffer at pH 6.8, being regularly shaken, at 25 °C. At predetermined times, the swelled beads and nanobeads were carefully withdrawn, the excess water was removed and the samples were weighed on an analytical balance [21] in triplicate for 6 h. The water uptake (g H_2_O/g sample) was calculated from Equation (2):

(2) $$\mathrm{Water}\;\mathrm{uptake}\;(g\;/\;g)\;=\frac{W_{2}-W_{1}}{W_{1}}$$

where *W*_2_ and *W*_1_ are the swelled and initial mass (0.05 g) of beads, respectively.

### 2.12. In Vitro Indomethacin Release Test

The beads and nanobeads (0.1 g) were immersed in 50 mL of the release medium in a thermostatic bath at 37 °C and under agitation (350 rpm). Considering the oral administration purpose, the samples were first maintained for 2 h in a 0.06 M HCl release medium (pH 1.2) to simulate conditions of the gastric tract. Then, they were kept at pH 6.8 (by adding 0.03 g NaOH and 0.40 g NaH_2_PO_4_·H_2_O), to mimic the intestinal microenvironment, until the end of the test (26 h). At predetermined time intervals, 1 mL of each solution was withdrawn, and INDO was quantified by UV-vis spectrophotometer (λ = 210 nm) [19]. The measured samples were returned to the release medium to keep the volume constant. All the experiments were carried out in quintuplicate. Data were given as the mean ± standard deviation of INDO cumulative release percentages.

KinetDS 3.0 software (Aleksander Mendyk, Kraków, Poland) was employed to analyze the release profile curves [22] of the beads and nanobeads, using several kinetic models: the zero order (Equation (3)) [23], Baker–Lonsdale (Equation (4)) [24] and Weibull (Equation (5)) models [25].

Q = k⋅t + Q_0_ (3)

3/2 [1 − (1 − Q)^2/3^] − Q = k·t (4)

where Q = INDO amount released at the time t, k = rate constant and Q_0_ = initial INDO release.

m = 1 − exp[−(t)^b^/a] (5)

where m = INDO concentration released at the time t, b gives the release exponent and a is the time scale of release.

### 2.13. In Vivo Toxicity Assays through Chicken Embryo Model

The in vivo toxicity of the INDO solution as the positive control (PC), the 0.9% NaCl solution as the negative control (NC) and the XAN@CHT/INDO (20 mg INDO/egg) bead and XAN@CHT/NLC-INDO (20 mg INDO/egg) nanobead was evaluated in a chicken embryo (CE) model through different parameters—embryos viability, CE weight and annexes changes—and blood biochemical markers. Eggs with 10 days of incubation (EID) were used. Then, the chorioallantoic membrane (CAM) was lowered, and the samples were administered in CAM (Figure S2) and followed for 7 days. After 24 h, the eventual CE deaths caused by the mechanical stress of CAM removal were excluded from the analysis.

### 2.14. Preparation of Eggs

The eggs of laying hens (Gallus gallus, Hy-Line W-36 lineage) were a gift of Hy-Line do Brazil (Uberlândia, Brazil). The eggs at 10 days of incubation (10 EID) were first carried out to a light ovoscopy, to confirm the successful embryonic development. Then, 48 eggs were weighted and incubated in an artificial incubator (Premium Ecológica^®^) at 37 °C and 58% relative humidity, being turned at a 2-hour interval up to subsequent analyses.

### 2.15. CE Viability Test

For the embryo mortality check, the eggshell was fragmented at 17 EID. The CE viability (%) was determined by the percentage of alive embryos with or without injuries, after 24 h of testing in comparison with the initial number of alive embryos (*n* = 12). The Chi-square test followed by binomial test between two proportions were used to elucidate intergroup significant differences (*α* = 0.05).

### 2.16. Changes in the Weights of CE and Annexes

At 17 EID, the CEs were weighed, and their annexes (CAM, egg yolk and amnion) were removed and also weighed (*n* = 12). The one-way ANOVA and Tukey post hoc tests were applied to elucidate intergroup statistically significant differences (*p* < 0.05). The weight of CE was adjusted based on the egg weight (EID = 10) according to the Equation (6):

aW = final^W^ × 50/initial^W^ (6)

where aW is the CE weight adjusted to 50 g, final^W^ is the CE weight at 17 EID and initial^W^ is the weight of embryonic egg at 10 EID.

### 2.17. Biochemical Markers

At 17 EID, the umbilical vessel blood from the CE was collected for biochemical analyses and analyzed in an automatic biochemical analyzer (ChemWell^®^ 2910, Awareness Technology, Palm, FL, USA). The analytes—uric acid (UA), alkaline phosphatase (ALP), creatine kinase (CK), gamma glutamyl transferase (GGT), alanine aminotransferase (ALT) and aspartate aminotransferase (AST) (Bioclin^®^, Minas Gerais, Brazil)—were quantified from the CE serum (*n* = 12). Data were expressed as median ± SEM. The one-way ANOVA/Tukey tests were performed for the analyses of intergroup differences of each marker.

## 3. Results

### 3.1. Preparation of Nanostructured Lipid Carriers Loading Indomethacin

Ten different NLC formulations (Table 1) composed of coconut oil (CO), myristyl myristate (MM) and poloxamer (P68), with and without INDO (2%), were successfully prepared, showing a homogenous aspect and pale white color.

### 3.2. Long-Term Stability of NLC

**Figure 1:**
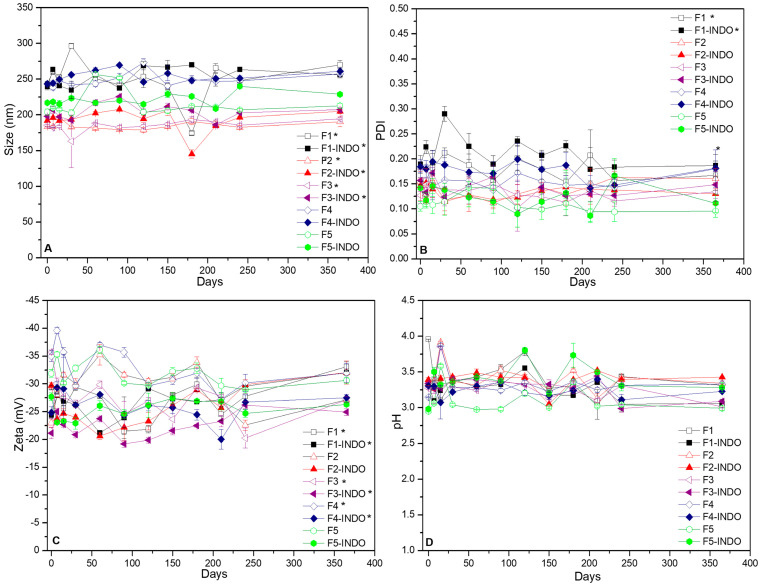
Long-term stability of NLC formulations, monitored in terms of size (**A**), PDI (**B**), Zeta potential (**C**) and pH (**D**) values for up to a year, at 25 °C (*n* = 3; *p* < 0.05).

The shelf life of the NLC formulations with or whitout 2% INDO were assessed through size (nm), polydispersity index (PDI), Zeta potential (mV), pH and visual inspection monitoring for 1 year at 25 °C (Figure 1). Visually, all the obtained colloid-like formulations were homogenous and white, with no phase separation during one year. As determined by DLS, the particle sizes of NLC formulations were smaller than 300 nm (Figure 1A). It is noteworthy that the F5 and F5-INDO formulations showed no statistically significant differences over time (*p* > 0.05). All systems showed desirable particle sizes after 1 year of storage. According to Figure 1B, all the prepared formulations exhibited PDI values lower than 0.2, throughout the stability monitoring.

Zeta potential values of formulations were in the range of −20 to −40 mV for the control (NLC) and NLC/INDO (Figure 1C) formulations, respectively. F5 and F5-INDO formulations showed no statistically significant differences (*p* > 0.05) in terms of Zeta values over 365 days. Finally, the pH values ranged from 3.0 to 3.5 for all samples (Figure 1D) and remained constant (*p* > 0.05) over the time.

### 3.3. Indomethacin Encapsulation Efficiency by NLC

INDO encapsulation efficiency by NLC was determined by the ultrafiltration–centrifugation method. Table 3 shows excellent (%EE very close to 100%) in all formulations tested.

Considering the absence of significant changes in the long-term stability and the %EE results, F5-INDO was chosen to be evaluated in further tests.

### 3.4. Transmission Electron Microscopy (TEM)

**Figure 2:**
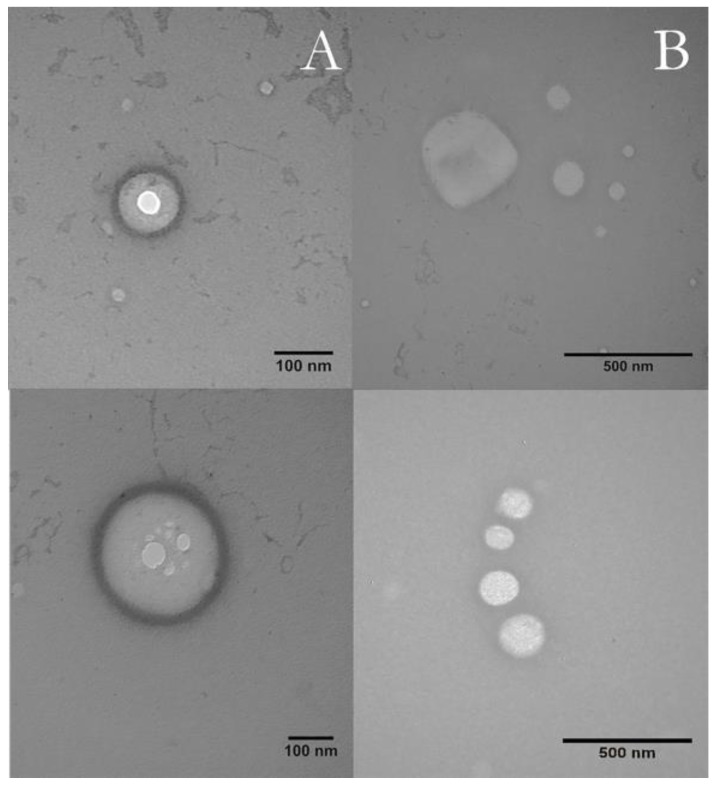
TEM micrographs (60 kV) of F5 (above) and F5 loading 2% indomethacin (F5-INDO) (below), at magnifications of 100,000× (**A**) and 60,000× (**B**).

Figure 2 shows the spherical morphology F5/INDO and F5 (as control) formulations, with well-defined boundaries. The particle sizes calculated from the micrographs using the ImageJ software agreed with those quantified by DLS (<300 nm).

### 3.5. Cell Viability Tests

**Figure 3:**
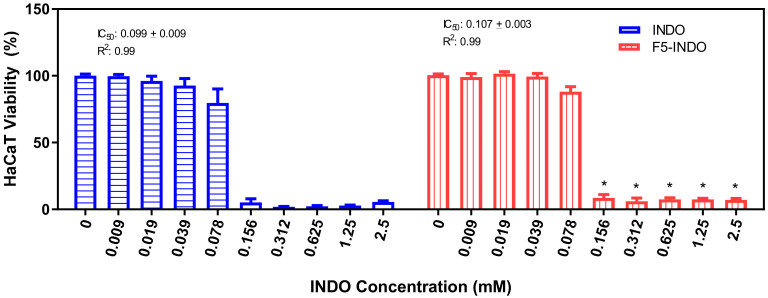
In vitro viability test in HaCaT cells (*n* = 9). T test determined statistically significant differences between free indomethacin (INDO) and F5-INDO formulations in different concentrations, (* *p* < 0.05).

The in vitro viability test (Figure 3) in HaCat cell line was performed to elucidate the cytotoxicity of INDO solution (control) compared with F5-INDO through the MTT test [26]. At the conditions tested, a significant increase (*p* < 0.05) in cell viability was registered when HaCat cells were treated with NLC-INDO (IC50 = 0.107 mM), in comparison to INDO solution (IC50 = 0.099 mM).

Considering all these results, NLC composed of 10% TL (MM = 80% and CO = 20%), 5% P68 and 2% INDO were selected as the best formulation, called from now on NLC-INDO, the lipid excipient of hybrid lipid–biopolymer nanobeads.

### 3.6. Preparation of Biopolymers and Hybrid Nanobeads Loading Indomethacin

**Figure 4:**
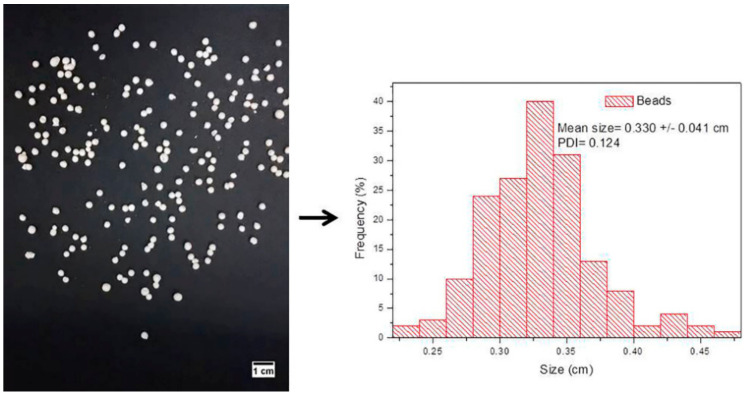
Digital photo (**left**) of hybrid nanobeads (XAN@CHT/NLC-INDO) and particle size distribution (**right**), estimated by ImageJ software, (*n* = 3).

The biopolymer beads (CHT/INDO and XAN@CHT/INDO) and hybrid nanobeads (CHT/NLC-INDO and XAN@CHT/NLC-INDO) were successfully prepared. Digital photos of all the samples (*data not shown*) were taken with at least 150 beads that were spatially dispersed. Then, the frequency of particle sizes from the images were estimated by ImageJ software, as exemplified in Figure 4, for XAN@CHT/NLC-INDO sample. Table 4 reveals that the particle sizes of beads were lower than 0.4 cm, with a monodisperse distribution (PDI < 0.130) for all the prepared samples.

### 3.7. FE-SEM Analyses

**Figure 5:**
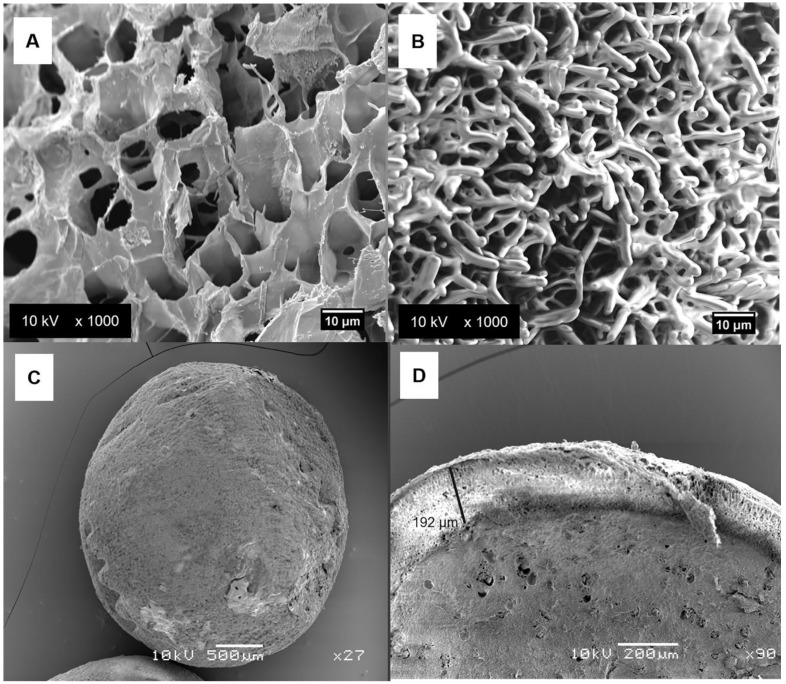
FE-SEM surface analyses of CHT/INDO (**A**) bead, CHT/NLC-INDO (**B**,**C**) and a cross-section of XAN@CHT/NLC-INDO (**D**) nanobeads. The figure emphasizes the thickness of xanthan coating, calculated by ImageJ software (**D**). The magnifications used were as follows: 1000× for (**A**,**B**), 27× for (**C**) and 90× for (**D**) images, operated at 10 kV. NOTE: CHT/INDO= chitosan bead loading INDO 2% (*w*/*v*), CHT/NLC-INDO= nanobead loading indomethacin 2% (*w*/*v*), XAN@CHT/NLC-INDO = xanthan-coated CHT/NLC-INDO nanobead.

Figure 5 displays the surface analyses of the different bead and nanobeads loading 2% INDO. The CHT/INDO (Figure 5A) and CHT/NLC-INDO (Figure 5B) images showed expected profiles of the flake-like chitosan structure [21] and rough surface of biopolymer–lipid assembling [27], respectively. The top-of-view of CHT/NLC-INDO (Figure 5C) shows the spherical and uniform morphology of the nanobead. Finally, the cross-section of XAN@CHT/NLC-INDO (Figure 5D) nanobead proves the successful coating of XAN, with a well-delimited external layer with around 192 µm of thickness. There was no evidence of dispersed drug in the biopolymer matrices in any of the images.

### 3.8. DSC Analyses

**Figure 6:**
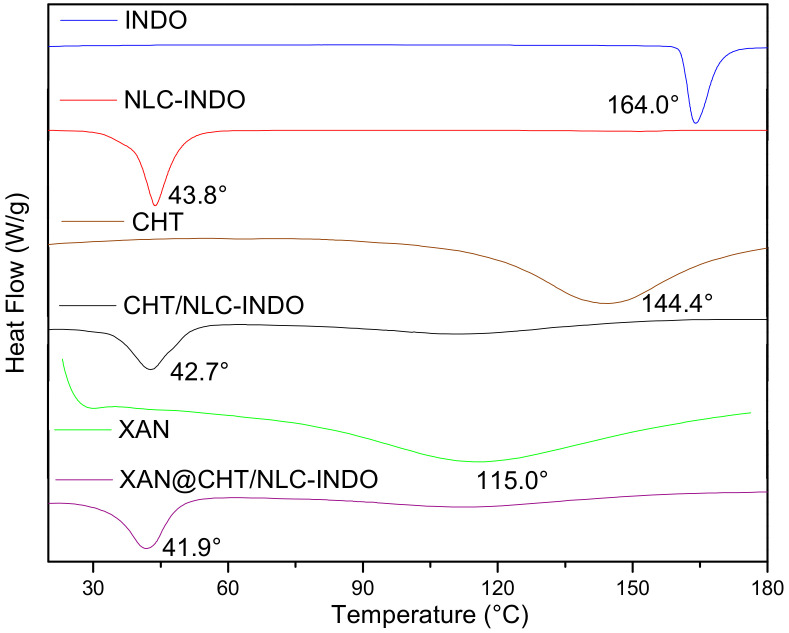
DSC thermograms for indomethacin (INDO), pure chitosan (CHT) and xanthan (XAN), as well as NLC-INDO formulation and CHT/NLC-INDO and XAN@CHT/NLC-INDO nanobeads.

The DSC thermodynamic profiles of the excipients and nanobeads are shown in Figure 6. Endothermic peaks corresponding to the melting points of INDO at 164.0 °C [28], NLC-INDO at 43.8 °C, CHT/NLC-INDO at 42.7 °C and XAN@CHT/NLC-INDO at 41.9 °C were detected. MM, in which melting point is at 36–42 °C [29], is the major component of NLC and nanobeads. In addition, two endothermic peaks, related to the biopolymers’ dehydration for CHT at 144.4 °C [30] and for XAN at 115.0 °C [15], were noticed.

### 3.9. In Vitro Water Uptake

**Figure 7:**
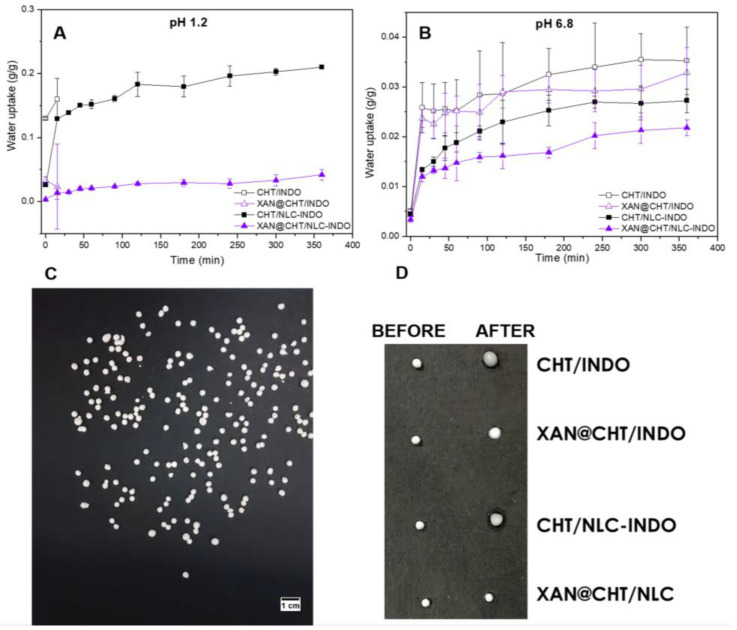
In vitro water uptake from beads and nanobeads in two different conditions: pH 1.2 (**A**) and pH 6.8 (**B**). Digital photos from the prepared samples (**C**) and the samples before and after the test (**D**) at pH 6.8 were provided. CHT/INDO = chitosan bead loading INDO 2% (*w*/*v*), XAN@CHT/INDO = xanthan-coated bead loading indomethacin 2% (*w*/*v*), CHT/NLC-INDO = nanobead loading indomethacin 2% (*w*/*v*), XAN@CHT/NLC-INDO = xanthan-coated CHT/NLC-INDO nanobead.

The swelling degrees of bead (CHT/INDO and XAN@CHT/INDO) and nanobeads (CHT/NLC-INDO and XAN@CHT/NLC-INDO) were assessed through the in vitro water uptake kinetics (Figure 7). It was performed at two different pH levels, simulating the gastric (pH 1.2) and intestinal (pH 6.8) microenvironments [23].

In acid medium, CHT/INDO and XAN@CHT/INDO beads swelled to 0.16 and 0.026 g, respectively, and disrupted after 15 min of experiment. On the other hand, the hybrid CHT/NLC-INDO and XAN@CHT/NLC-INDO nanobeads did not disrupt, and swelled to 0.21 and 0.042 g, respectively, after 360 min. The water uptake in the simulated intestinal system (pH 6.8) evidenced that all beads were visually intact after 6 h (see the digital photo of all beads and nanobeads, before and after the experiment, in Figure 7). Moreover, the final water uptake of CHT/INDO, XAN@CHT/INDO, NLC-INDO and XAN@CHT/NLC-INDO was 0.035, 0.033, 0.027 and 0.022 g, respectively.

### 3.10. In Vitro Indomethacin Release Test

**Figure 8:**
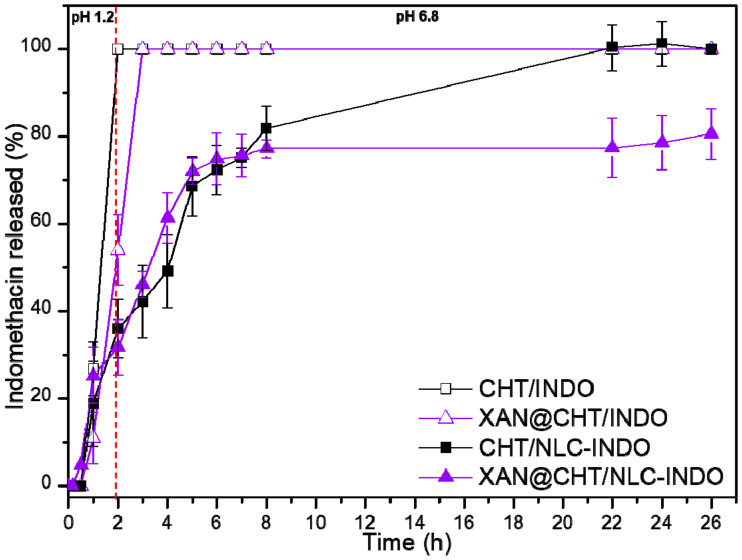
Indomethacin in vitro release from beads (CHT/INDO and XAN@CHT/INDO) and nanobeads (CHT/NLC-INDO and XAN@CHT/NLC-INDO), quantified by UV-vis (λ = 210 nm) at 37 °C, *n* = 5.

The in vitro INDO release test was conducted for 26 h at 37 °C (Figure 8). In the first 2 h, the experiment simulated the gastric system (pH 1.2). Then, the pH of the external medium was changed to 6.8, mimicking the intestinal system [21] until the end of the analysis.

CHT/INDO and XAN@CHT/INDO beads exhibited burst effects, resulting in total (100%) INDO release in the second and third hour of the experiment, respectively. Differently, in the first 2 h of analysis, CHT/NLC-INDO and XAN@CHT/NLC-INDO nanobeads released around 36% and 31%, respectively. CHT/NLC-INDO released 100% of the anti-inflammatory after 22 h, while XAN@CHT/NLC-INDO released around 80% at the end of the experiment (26 h).

Mathematical modeling of the kinetics was carried out by KinetD software [22]. Different models were tested and considering the highest coefficient of determination (R^2^; Table 5), the beads (CHT/INDO and XAN@CHT/INDO) were best fitted by the zero-order model, while CHT/NLC-INDO and XAN@CHT/NLC-INDO hybrid nanobeads were best fitted by the Baker–Lonsdale and Weibull models, respectively.

### 3.11. In Vivo Toxicity Assay through Chicken Embryo Model

**Figure 9:**
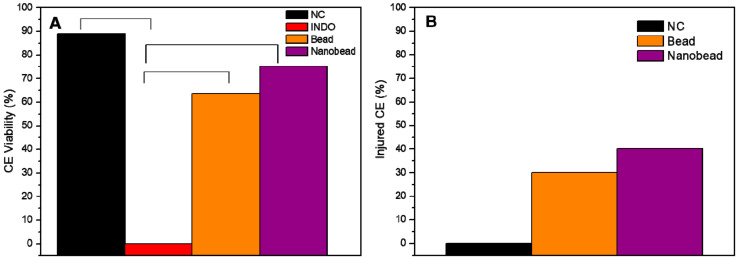
In vivo toxicity test through CE model in terms of embryos viability (**A**) and injured CE (**B**) after treatments with 0.9% NaCl solution (NC), indomethacin solution (20 mg/egg) as INDO, xanthan-coated chitosan bead loading indomethacin (bead) (XAN@CHT/INDO; 20 mg/egg) and xanthan-coated nanobead (nanobead) (XAN@CHT/NLC-INDO; 20 mg/egg). The Chi-square test, followed by binomial test between two proportions, was performed to compare INDO (**A**) or NC (**B**) with the bead or nanobead, considering their respective contingency tables (2 × 2). Statistically significant differences (*p* < 0.05) are shown in (**A**), *n* = 12. (**B**) shows descriptive results.

The alternative in vivo toxicity assay through CE model [31] was used to evaluate the toxicity of INDO (in solution) in comparison with the coated bead (XAN@CHT/INDO) and nanobead (XAN@CHT/NLC-INDO). Figure 9 shows the CE viability (%) after treatment with the different formulations. Since all CE treated with INDO (as positive control) died during the experiment, the viability percentages of other groups were statistically different (*p* < 0.05). On the other hand, there was no significant differences (*p* > 0.05) in the viability of CE treated with beads and nanobeads, in comparison to the negative control, NC (Figure 9A). The percentages of the CE viability for beads and nanobeads were 60% and 80%, respectively. Among the CE survivors, approximately 30% treated with beads and 40% with nanobeads induced injury to the embryos (Figure 9B).

**Figure 10:**
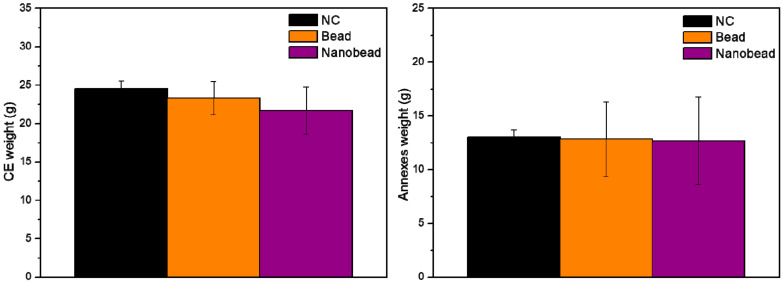
The weight (g) of chicken embryo (CE) and annexes after 48 h of treatments with 0.9% NaCl solution (NC), xanthan-coated bead loading indomethacin (bead) (XAN@CHT/INDO; 20 mg/egg) and xanthan-coated nanobead (nanobead) (XAN@CHT/NLC-INDO; 20 mg/egg). Data are expressed as mean ± SEM, *n* = 12. One-way ANOVA/Tukey tests were carried out for intergroup statistically differences analyses (*p* < 0.05). INDO treatment was not considered once there was no survivors after 48 h.

The weights of CE and annexes treated with NC, beads and nanobeads containing INDO (20 mg/egg) are given in Figure 10. In terms of CE weight, it was noticed values of 24.53, 23.31 and 21.71 g when treated with NC, beads and nanobeads, respectively. On the other hand, the weights of the annexes were 13.05, 12.82 and 12.68 g for NC, beads and nanobeads, respectively. There were no statistically significant differences among the CE (*p* > 0.05) and annexes (*p* > 0.05) weights in comparison with NC.

After 48 h, the CE were euthanized and the levels of biochemical markers in the serum were provided in terms of UA (mg/dL), CK (U/L), ALP (U/L), GGT (U/L), AST (U/L) and ALT (U/L). Table 6 displays the values of all analytes of CE treated with NC, beads and nanobeads. Among all the enzyme activities, only the ALP values were statistically significant different (higher) to the NC (*p* < 0.05). For all the other markers, no statistically significant differences were observed.

## 4. Discussion

The oral route is the major choice of drug administration [32]. After absorption, the actives are subjected to hepatic first-pass metabolism, which can result in low efficacy and side effects, mainly to the gastrointestinal system [33]. That is the case of INDO, a NSAID largely prescribed for the management of chronic pain and inflammatory conditions. Side effects are even more accentuated in prolonged therapies, decreasing the patient compliance [2].

The current work was divided in two parts: the development and characterization of nanostructured lipid carriers (NLC-INDO), followed by the preparation and evaluation of beads (XAN@CHT/INDO) and nanobeads (XAN@CHT/NLC-INDO).

Different NLC formulations (10) exhibited excellent in vitro structural properties, reinforcing such systems as promising DDS. INDO encapsulation by NLC caused slight changes in the biophysical parameters, preserving the morphology, size homogeneity and nanoparticles steric repulsion over time, as required for a long-term stability. In fact, such formulations showed lower pH values than control (without drug), given to the INDO acid character [24] and high amount of INDO upload by NLC/INDO (close to 100%), as expected for hydrophobic NSAID [6,34].

F5-INDO formulation confirmed the decrease in the INDO cytotoxicity in comparison with free INDO treatment. Unfortunately, the oral administration of colloid systems is contraindicated, given their poor gastro-resistance. It is worth mentioning that the prepared NLC-INDO can be further explored as DDS aiming other administration routes, such as parenteral, intranasal and/or transcorneal, for the sustained delivery of INDO. Therefore, F5-INDO was selected as the lipid component (called as NLC/INDO) in the nanobeads development.

Lipid–polymer assembling is a remarkable combination of organic matrices resulting in hybrid DDS with extraordinary properties and complex supramolecular arrangements [35,36,37]. In here, the first step in the development of the lipid–biopolymer nanobead consisted in the CHT/NLC-INDO preparation. Such a system was intended to combine the advantages of INDO nanoencapsulation by NLC with the deeply explored mucoadhesion property of cationic CHT [13]. The challenge of the second step was to protect the resultant hybrid nanobead against the acidic stomach pH, once both excipients—CHT and NLC/INDO—were not gastro-resistant (a requirement to avoid fast absorption and first-pass metabolism). In this sense, the enteric coating of beads, microspheres, tablets and colloids to provide gastro-resistance to the systems is not a novelty. Different coatings based on pectin, alginate, carboxymethylcellulose, eudragit, gelatin, gellan and xanthan gums, among others, avoided drug release in simulated stomach condition successfully [10,21,38,39]. In here, XAN, which is a rheological improver [15], coated CHT/NLC-INDO. XAN acted as a physical barrier, preventing CHT/NLC-INDO degradation in acid conditions, processed as XAN@CHT/NLC-INDO (coated nanobead). The advantages of XAN@CHT/NLC-INDO in comparison with the non-coated related nanobead (CHT/NLC-INDO) and beads (CHT/INDO and XAN@CHT/INDO) are emphasized below.

From the structural point of view, the particle size distribution of the nanobeads (Figure 4) did not increase in relation to pure biopolymer forms (Table 3). In addition, FE-SEM images successfully confirmed the XAN coating procedure, with a well-delimited and homogenous external layer (Figure 5D), acting as physical barrier to protect CHT and NLC-INDO to the degradation. Moreover, calorimetric analyses suggested good compatibility between the lipid–biopolymer blend and INDO, since the thermodynamic properties of XAN@CHT/NLC-INDO are very similar to that of NLC-INDO, the major component of nanobeads [15,40]. Still, no degradation peaks were observed in the thermograms, ensuring the thermal stability of the system up to 180 °C (Figure 6), far above the physio/pathological temperature range (32.5–41.0 °C).

The hybridization and coating procedures were responsible to modulate the in vitro performance of nanobeads, regarding water uptake (Figure 6) and INDO release (Figure 8) kinetics tests. The first two hours of both experiments were conducted in acid condition (pH 1.2) simulating the gastric pass, and the subsequent hours were conducted in pH 6.8, simulating the intestinal microenvironment. CHT/INDO and XAN@CHT/INDO beads were disrupted after 15 min and released 100% of INDO in acid medium, due to their solubilization. Both nanobeads (CHT/NLC-INDO and XAN@CHT/NLC-INDO) sustained INDO release in pH 1.2, remaining intact until the end of water uptake test (pH 6.8), and exhibiting an INDO prolonged release profile. The lipid counterpart in the nanobeads increased the hydrophobicity [41] and also prolonged INDO delivery.

Despite that XAN@CHT/NLC-INDO prolonged INDO release up to 26 h, the desirable burst release effect (~30%) was also noticed in the first two hours of test. This is due to the amount of INDO directly incorporated in the CHT matrix (to reach the drug final concentration of 2%), allowing for its faster release. In general, a burst effect followed by a prolonged release profile are essential for DDS aiming pain and inflammation management [42]. Specifically, XAN exerted a physical protection to the nanobead (XAN@CHT/NLC-INDO) and bead (XAN@CHT/INDO) that decreased CHT and NLC/INDO solubilization in acid in comparison with their respective non-coated forms. Still, XAN@CHT/NLC-INDO, which combined the hybridization and coating processes, exhibited the lowest water uptake and a higher INDO sustained release profile among all the samples. This inverse relation between swelling and release profiles is expected for hybrid polymer-based forms, once a higher resistance to water adsorption is correlated with a more sustained profile of entrapped drugs [21,23]. The prolonged INDO delivery was explained by the two physical barriers that INDO had to overcome, the lipid structural matrix and the swelled biopolymers chains of XAN@CHT/NLC-INDO [15,27]. In fact, the differences of XAN@CHT/INDO (as bead) and XAN@CHT/NLC-INDO (as nanobead) swelling capacity were also observed in the vivo assays, where the hybrid nanobead was intact after 7 days of CE administration, while the beads were totally solubilized at this time (Figure S2).

It is also interesting to notice that the mathematical modeling indicated that the novel supramolecular arrangement of nanobeads was also responsible for different INDO kinetics mechanisms. Both biopolymer beads were best fitted by the zero-order model, as expected, considering their total INDO release up to 2–3 h of the test. On the other hand, CHT/NLC-INDO was best fitted by the Baker and Lonsdale model, which describes a sustained diffusion of drug from a spherical matrix [43]. Besides, XAN@CHT/NLC-INDO was best fitted by Weibull model, indicating a biphasic profile from complexes matrices, given by the release exponent values (b < 1). The nanobead combined a burst effect with prolonged release INDO profile. In fact, the Weibull model is widely employed to explain the complexes lipid–polymer DDS kinetics mechanisms [15,40,41,44].

Finally, the safety of DDS is another mandatory requisite, especially in case of nanomaterials that exhibit novel kinetics and supramolecular arrangements, to detect possible risk factors to health [45]. In this sense, CE is an alternative toxicity model that is very promising to evaluate DDS, once different parameters (microscopical, microscopical and biochemical) are evaluated in a single model [31]. In here, the CE viability test showed that INDO solution (PC) killed all embryos after 24 h, which was not observed for bead and nanobead (Figure 9A) treatments. Moreover, the indexes of surviving embryos of NC did not show statistically significant differences (*p* > 0.05) compared to the groups treated with beads or nanobeads. In relation to the changes in the CE and annexes weight, significant differences among the groups and NC were not detected (*p* > 0.05).

Furthermore, the analysis of biochemical parameters provides essential information for the evaluation of animal global health status. All the metabolites of CE treated with bead and nanobead did not significantly change in comparison with NC (*p* > 0.05), except for the ALP values of CE treated with beads (*p* < 0.05). In fact, there is some evidence that INDO can induce an increase in ALP in rats, indicating its ability to induce toxicity to the liver [46]. It is worth mentioning that the CE treated with beads, where INDO was only dispersed in CHT matrix, resulted in a liver injury, given by ALP increase and macroscopic changes in liver. Interestingly, the treatment with nanobeads showed less ability to damage the CE liver than beads. This is probably due to the different lipid–biopolymer barriers and the nanoencapsulation that decreased INDO toxicity in CE together with the biopolymer protective effect. Such evidence suggests that the nanobead was the least toxic treatment for CE, not causing significant deaths, macroscopic damages or biochemical markers alterations to CE.

These results strongly suggested that XAN@CHT/NLC-INDO was effective to prolong the INDO release profile, exhibited gastro-resistant properties and decreased the drug toxicity, as claimed. Such a system can be a promising candidate as an INDO oral delivery system, and should be further tested in efficacy assays.

## 5. Conclusions

This work described the preparation of elegant hybrid nanobeads composed of nanostructured lipid carriers loading indomethacin (NLC/INDO; 2%) and chitosan (CHT; 2%), coated by xanthan (XAN; 0.5%). Such systems presented excellent structural and thermodynamic properties, in vitro swelling properties and a prolonged drug release profile up to 26 h. The synergism between the nanolipid and biopolymer excipients allowed to prevent the burst release effect in the first 2 h of experiments, simulating the gastric medium, followed by a prolonged release in the pH 6.8, mimicking the intestinal medium. The XAN coating of nanobeads (XAN@CHT/NLC-INDO) acted as a gastro-resistant excipient protecting NLC/INDO and CHT from an abrupt swelling and consequent total drug release in the first hours of experiments. The in vivo toxicity assay in chicken embryos (CE) confirmed the nanobeads safety, which combined the advantages of the hybridization and coating procedures, decreasing the intrinsic toxicity of INDO in CE in all the analyzed parameters. Overall, the lipid–biopolymer nanobead proposed here is an excellent candidate for the delivery of INDO, as well as other anti-inflammatories through the oral route.

## Figures and Tables

**Table 1:** Composition of NLC formulations.

Formulation	INDO (%)	MM:CO (%)	P68 (%)
F1	0	80:20	2
F1/INDO	2	80:20	2
F2	0	70:30	5
F2/INDO	2	70:30	5
F3	0	60:40	5
F3/INDO	2	60:40	5
F4	0	70:30	2
F4/INDO	2	70:30	2
F5	0	80:20	5
F5/INDO	2	80:20	5

NOTE: MM = myristyl myristate (solid lipid), CO = coconut oil (liquid lipid), MM:CO = ratio of solid and liquid lipids, P68 = poloxamer F68 (surfactant). NLC were prepared with 10% total lipid (MM + CO; *w/w*), the percentages are related to the weight/volume.

**Table 2:** Composition of the prepared beads and nanobeads encapsulating 2% indomethacin.

Sample	Composition	Form
CHT/INDO	Chitosan, indomethacin	Bead
XAN@CHT/INDO	Xanthan, chitosan and indomethacin	Coated Bead
CHT/NLC-INDO	Chitosan, nanostructured lipid carrier loading indomethacin	Nanobead
XAN@CHT/NLC-INDO	Xanthan and chitosan, nanostructured lipid carrier loading indomethacin	Coated Nanobead

**Table 3:** Encapsulation efficiency from different NLC formulations loading indomethacin (2%), *n* = 3.

Formulations	%EE
F1	-
F1-INDO	98.7 ± 0.2
F2	-
F2-INDO	99.1 ± 0.1
F3	-
F3-INDO	98.9 ± 0.8
F4	-
F4-INDO	99.0 ± 0.7
F5	-
F5-INDO	99.0 ± 0.2

**Table 4:** Frequency of size (cm) and distribution (PDI) of the prepared beads and nanobeads, obtained from digital photos and calculated by ImageJ software, *n* = 3.

Bead	Size (cm)	PDI
CHT/INDO	0.398 ± 0.009	0.112 ± 0.001
CHT/NLC-INDO	0.370 ± 0.004	0.102 ± 0.006
XAN@CHT/INDO	0.352 ± 0.011	0.110 ± 0.040
XAN@CHT/NLC-INDO	0.330 ± 0.021	0.124 ± 0.009

NOTE: CHT/INDO = chitosan bead loading INDO 2% (*w*/*v*), CHT/NLC-INDO = nanobead loading indomethacin 2% (*w*/*v*), XAN@CHT/INDO = xanthan-coated bead loading indomethacin 2% (*w*/*v*), XAN@CHT/NLC-INDO = xanthan-coated CHT/NLC-INDO nanobead.

**Table 5:** Mathematical modeling of release kinetics, fitted with several mathematical models. The values displayed corresponds to the coefficient of determination (R^2^), calculated by KinetD software. The highest R^2^ for each group are in bold.

Formulation	0 Order	Weibull	Baker–Lonsdale
CHT/INDO	**0.95**	0.78	0.83
XAN@CHT/INDO	**0.97**	0.82	0.75
CHT/NLC-INDO	0.66	0.74	**0.99**
XAN@CHT/NLC-INDO	0.46	**0.91**	0.59

NOTE: CHT/INDO = chitosan bead loading INDO 2% (*w*/*v*), CHT/NLC-INDO = nanobead loading indomethacin 2% (*w*/*v*), XAN@CHT/INDO = xanthan-coated bead loading indomethacin 2% (*w*/*v*), XAN@CHT/NLC-INDO = xanthan-coated CHT/NLC-INDO nanobead.

**Table 6:** Biochemical markers indexes in the CE serum treated with different groups. Data are provided as median ± SEM, n = 12. One-way ANOVA/Tukey tests were performed for the analyses of intergroup differences among each marker. ^a,b^ = (*p* < 0.05).

Markers	NC	Bead	Nanobead
UA (mg/dL) CK (U/L)	31.70 (±15.2) 1356.0 (±1494.0)	18.97 (±12.6)	50.69 (±43.4)
		513.1 (±501.1)	582.4 (±420.9)
ALP (U/L)	429.6 (±230.4) ^a^	1168.0 (±454.8) ^b^	946.7 (±449.2) ^ab^
GGT (U/L)	6.98 (±4.9)	6.52 (±3.7)	5.28 (±4.3)
AST (U/L)	144.6 (±146.4)	118.6 (±31.4)	153.8 (±94.0)
ALT (U/L)	11.40 (±8.0)	5.80 (±3.3)	4.60 (±5.7)

NOTE: NC = 0.9% NaCl, bead = xanthan-coated chitosan bead loading indomethacin (XAN@CHT/INDO; 20 mg/egg) and nanobead = xanthan-coated nanobead (XAN@CHT/NLC-INDO; 20 mg/egg).

## Data Availability

The data presented in this study are available on request from the corresponding author.

## Supplementary Materials

The following supporting information can be downloaded at: https://www.mdpi.com/article/10.3390/pharmaceutics14030583/s1, Figure S1: Indomethacin calibration curve (detection at 210 nm) performed on five different concentrations in the range of 3.25–20 µg/mL. Correlation coefficient was >0.99. Each point represents the average of nine measurements performed in three different days; Figure S2: Digital photos of the chicken embryos treated with beads (above) and nanobeads (below) in different times of incubation.
