# Supplementary material for: Hybrid Nanobeads for Oral Indomethacin Delivery
Source: Pharmaceutics. 2022 Mar 8;14(3):583. doi: 10.3390/pharmaceutics14030583 (PMC8954626; doi:10.3390/pharmaceutics14030583)
Supplement: Supplementary file 1 [file pharmaceutics-14-00583-s001.zip › pharmaceutics-1574814-supplementary.pdf]

pharmaceutics

MDPI

Supplementary Materials: Hybrid Nanobeads for Oral Indomethacin Delivery

Flávia Monique Rocha Bonetti, Eneida de Paula, Belchiolina Beatriz Fonseca, Gabriela Ribeiro da Silva, Leandro Santana Soares da Silva, Ludmilla David de Moura, Márcia Cristina Breitkreitz, Gustavo Henrique Rodrigues da Silva and Lígia Nunes de Morais Ribeiro

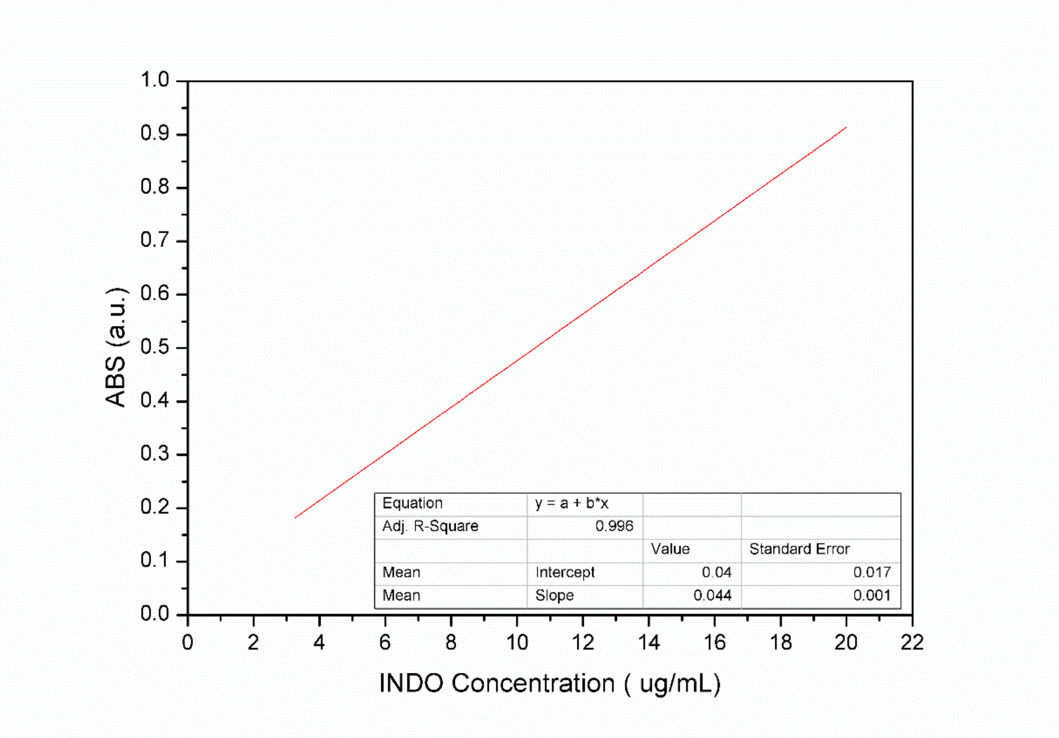

Figure S1. Indomethacin calibration curve (absorbance measured at 210 nm) performed on five different concentrations in the range 3.25–20 µg/mL. Correlation coefficient was >0.99. Each point represents the average of 9 measurements performed in 3 different days.

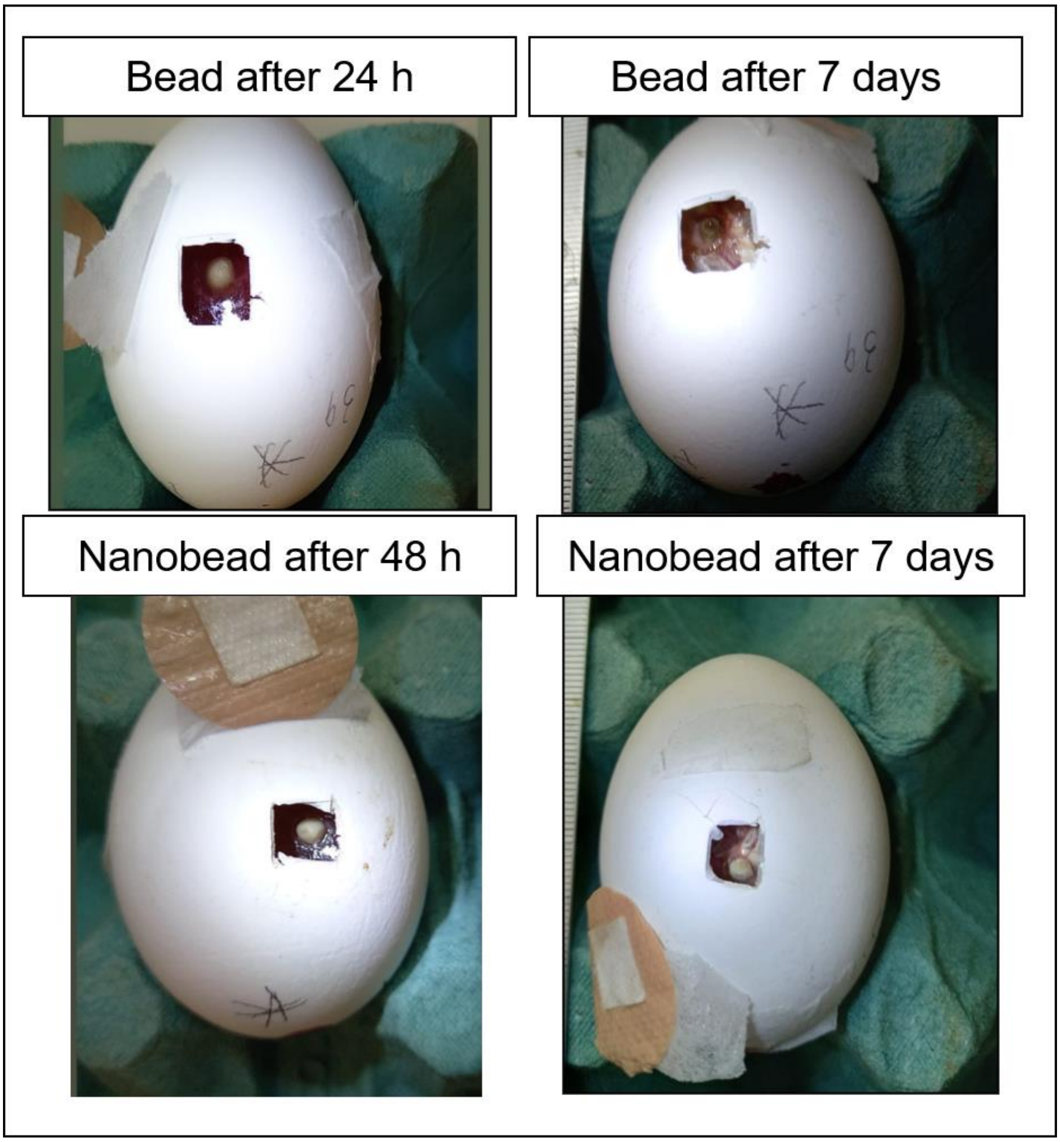

Figure S2. Digital photos of the chicken embryos treated with Beads (above) and Nanobeads (below) in different times of incubation.
